# Predictive values of upper gastrointestinal cancer alarm symptoms in the general population: a nationwide cohort study

**DOI:** 10.1186/s12885-018-4376-8

**Published:** 2018-04-18

**Authors:** Sanne Rasmussen, Peter Fentz Haastrup, Kirubakaran Balasubramaniam, René DePont Christensen, Jens Søndergaard, Dorte Ejg Jarbøl

**Affiliations:** Research Unit of General Practice, Department of Public Health, University of Southern Denmark, J. B. Winsløws Vej 9A, 5000 Odense C, Denmark

**Keywords:** Oesophagogastric cancer, Symptom, Diagnosis, Primary health care, Survey

## Abstract

**Background:**

Survival rates for upper gastrointestinal (GI) cancer are poor since many are diagnosed at advanced stages. Fast track endoscopy has been introduced to prompt diagnosis for patients with alarm symptoms that could be indicative of upper GI cancer. However, these symptoms may represent benign conditions and little is known about the predictive values of alarm symptoms of upper GI cancer in the general population.

**Methods:**

The study is a nationwide cohort study of 60,562 individuals aged 45 years or above randomly selected from the Danish general population. Participants were invited to complete a survey comprising of questions on several symptom experiences, including alarm symptoms for upper GI cancer within the past four weeks. The participants were asked about specific symptoms (repeated vomiting, difficulty swallowing, signs of upper GI bleeding or persistent and recent-onset abdominal pain) and non-specific symptoms (nausea, weight loss, loss of appetite, feeling unwell and tiredness).

We obtained information on upper GI cancer diagnosed in a 12-month period after completing the questionnaire from the Danish Cancer Registry. We calculated positive predictive values and positive likelihood ratios for the association between alarm symptom and subsequent upper GI cancer.

**Results:**

A total of 33,040 individuals above 45 years completed the questionnaire, yielding a response rate of 54.6%. Respondents were fairly respresentative of the study sample. During the follow-up period, 18 people were diagnosed with upper GI cancer. The number of incident cancers was similar among eligible non-respondents. Two thirds of the respondents with an upper GI malignancy had experienced one or more alarm symptoms.

The positive predictive value for being diagnosed with upper GI cancer after reporting a least one alarm symptom was 0.1% (95% CI:0.0–0.1%). The positive likelihood ratio was 4.4 for specific alarm symptoms and 1.1 for non-specific alarm symptoms.

**Conclusions:**

We found that positive predictive values of alarm symptoms of upper GI cancer experienced in the general population are low. It is important knowledge that despite denoted alarm symptoms even patients with specific alarm symptoms of upper GI cancer have a low risk of being diagnosed with upper GI cancer.

## Background

The incidence of upper gastrointestinal (GI) cancer comprising oesophageal and gastric carcinomas is modest in the Western world. However, upper GI cancers are serious diseases with substantial morbidity and mortality. The prognosis depends on the stage of disease at diagnosis and since many are diagnosed at advanced stages, the five-year survival rates are poor [1]. Approximately one third of the patients with upper GI cancer are diagnosed following emergency presentation [2], which is associated with poorer outcomes [3]. Referral guidelines for fast track endoscopy have been implemented to expedite diagnosis in order to avoid emergency presentation and to diagnose these cancers at less advanced stages [4, 5]. The guidelines give access to fact track endoscopy for patients presenting specific alarm symptoms indicative of upper GI cancer such as blood in vomit and new onset dyspepsia in individuals over 45 years. We have previously reported that specific alarm symptoms of upper GI cancer are not very prevalent in the general population [6]. Much of the evidence supporting referral guidelines derive from secondary care settings and patients already diagnosed with upper GI cancer. Previous studies in the primary care population have found the diagnostic performance of specific alarm symptoms to be rather poor [7]. Moreover, it has been demonstrated that almost half of cancer patients in general report unspecific symptoms prior to diagnosis [8]. Hence, the non-specific symptoms of malignant disease such as unintended weight loss and tiredness/fatigue are also important to take into consideration in the diagnostic process. Although the predictive values of specific and non-specific alarm symptoms of upper GI cancer in the general population are assumed to be low, this has not yet been investigated. It might be possible that healthcare seeking with alarm symptoms is associated with a higher likelihood of being diagnosed with a serious disease. However, knowledge about the predictive value of upper GI cancer alarm symptoms presented to the GP remains yet to be explored in a prospective study in the general population.

## Methods

### Aim

The aims of this study were 1) to determine the predictive value of specific and non-specific alarm symptoms for subsequent upper GI cancer in the general population over 45 years and 2) to describe the proportion of specific and non-specific alarm symptoms reported by patients prior to diagnosis of upper GI cancer. The duration of follow-up was 12 months.

### Study design and population

The study was designed as a nationwide cohort study based on questionnaires and national registries, imbedded in the Danish Symptom Cohort (DaSC) [9]. From the Danish Civil Registration System (CRS), 100,000 adults aged 20 years or above were randomly selected and invited to participate in a survey. All Danish citizens are registered in the CRS with a unique personal identification number. Prior to the sampling procedure, individuals who had indicated that they did not want research-related inquiries were excluded. Selected individuals received a postal letter explaining the purpose of the study. The questionnaire was designed using the internet-based platform SurveyXact [10]. In the letter a unique 12-digit login for a secure webpage was included. This provided access to a comprehensive web-based questionnaire. In order to prevent exclusion of people with no access to the internet, the participants were offered to complete the survey by telephone interview. When an invited subject was unable to respond due to severe illness or having moved abroad, family or relatives could decline the invitation on behalf of the invited person. The reason for not responding was then simply registered as illness or moved abroad.

### The questionnaire

We developed a comprehensive questionnaire on specific and non-specific cancer alarm symptoms. The questionnaire was based on standard rating scales, previously validated questionnaires and ad hoc items. The methodological framework for developing, piloting and field-testing the questionnaire is described elsewhere [9]. This paper addresses the specific and non-specific alarm symptoms that might indicate upper GI cancer. Symptoms were selected based on a review of literature, national and international cancer referral guidelines and descriptions of cancer pathways [4, 5]. In total, nine predefined symptoms form the base of this paper (Table 1).

**Table 1:** Specific and non-specific symptoms of upper gastrointestinal (GI) cancer

Specific alarm symptoms of upper GI cancer	Prevalence estimates of the symptoms (n (%)), *N* = 33,040
Repeated vomiting	350 (1.1%)
Difficulty swallowing	1119 (3.4%)
Upper GI bleeding^a^	454 (1.4%)
Persistent and recent-onset abdominal pain^b^	832 (2.5%)
Non-specific alarm symptoms of upper GI cancer	
Nausea	3040 (9.2%)
Weight loss	859 (2.6%)
Loss of appetite	1586 (4.8%)
Feeling unwell	3502 (10.6%)
Tiredness	13,745 (41.6%)

^a^Upper GI bleeding comprises experiencing ‘Blood in vomit’ and ‘Black stool’

^b^‘Persistent and recent-onset abdominal pain’ comprises experiencing ‘Abdominal pain’ for the first time more than one month ago but less than six months ago

Respondents were asked whether they had experienced one or more of the symptoms within the preceding four weeks. Further, they were asked whether they had contacted their GP regarding the symptom. The wording of the question regarding symptom experience was: “Have you experienced any of the following sensations, symptoms or discomfort within the past four weeks?”. Respondents select one or more of the pre-defined symptoms. With regard to GP contact, the question was worded: “Have you contacted your general practitioner concerning the symptom(s) you have experienced within the preceding four weeks, by appointment, telephone or e-mail?” An item concerning when the symptom(s) occurred for the first time was also included. The response categories were: “Less than one month ago”, “1-3 months ago”, “3-6 months ago” or “more than six months ago”.

### Register data

Information on diagnoses of upper GI cancer was retrieved from the Danish Cancer Registry (DCR). The DCR contains personal and tumour characteristics for all incident cancer cases in Denmark including date of diagnosis and ICD-10 codes for the lesions [11] . Only cases diagnosed in a 12-month period after the completion of the questionnaire were included. Furthermore, cases were excluded if the individual had been diagnosed with the same ICD-10 code in a time period covering five years prior to the completion of the questionnaire. The ICD-10 codes used for this study are listed in Table 2.

**Table 2:** ICD10 codes used for incident cancer cases

ICD10 diagnose code	Name
DC15 + DC16	Neoplasma malignum oesophagi + Neoplasma malignum ventriculi
DC150-DC159, excl. DC159X DC160-DC169 excl. DC169X	Malignant neoplasm in various parts of esophagus and stomach.

### Statistical analyses

Positive predictive values (PPVs) were calculated by dividing the number of symptomatic individuals diagnosed with upper GI cancer by the total number of symptomatic individuals in each category and are presented as percentages. PPVs for upper GI cancer were calculated for: 1) at least one of the nine alarm symptoms, 2) at least one of the specific alarm symptoms, 3) at least one of the non-specific alarm symptoms and 4) GP contact with at least one of the nine alarm symptoms. We chose to include positive likelihood ratios (LR+) as a relative measure of the association between symptom experience and upper GI cancer. This was done because we expected that the incidence of upper GI cancer would be low compared to the incidence of upper GI symptoms. Therefore, the numerator would be much smaller than the denominator when calculating the PPVs, running the risk of attenuating the association between symptom experience and upper GI cancer. The proportions of alarm symptoms reported by individuals diagnosed with upper GI cancer are presented. Confidence intervals were calculated using a binomial distribution.

All statistical tests used a significance level of *P* < 0.05. Data analyses were conducted using STATA statistical software 13.1 (StataCorp, College Station, TX, USA).

## Results

**Fig. 1:**
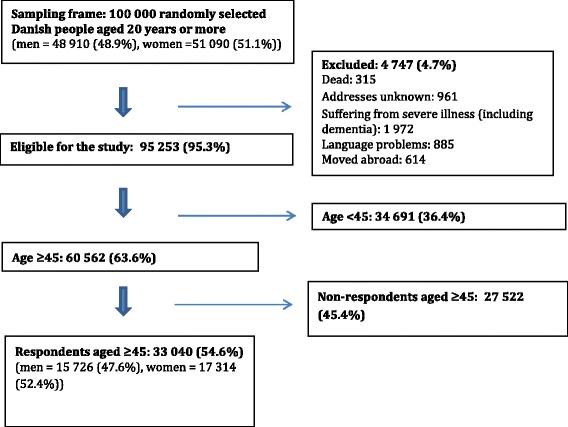
Study cohort

Of the 100,000 randomly selected subjects, 4474 (4.7%) were not eligible because they had either died, could not be reached due to an unknown address, were suffering from severe illnesses (including dementia), had language problems or had moved abroad. A total of 95,253 subjects were eligible for the study, of these 60,562 were ≥ 45 years of age. Of the 60,562 subjects, 33,040 completed the questionnaire, yielding an overall response rate of 54.6% (Fig. 1). The median age of the respondents in the group of respondents above 45 years was 60 years (interquartile range 52–68) compared to 63 years (interquartile range 53–73) for non-respondents. Slightly more respondents were women (52.4%) compared to non-respondents (51.0%). Details of the responder analysis of the questionnaire is described elsewhere [12].

We found that the specific alarm symptoms of upper GI cancer were infrequent in the general population (Table 1). ‘Difficulty swallowing’ was the most frequently reported symptom, reported by 1119 (3.4%) of the respondents. The non-specific alarm symptoms were more common. ‘Tiredness’ was reported by 13,745 (41.6%) of the respondents.

In total, 18 respondents were diagnosed with upper GI cancer within the 12 month follow-up period after questionnaire completion. The number of incident upper GI cancers was similar among eligible non-respondents (14/27,522). The median age of the respondents diagnosed with upper GI cancer was 69.0 years.

The PPVs for being diagnosed with upper GI cancer ranged between 0.1% (95%-CI: 0.0–0.1) for at least one of the nine specific and non-specific cancer alarm symptoms and 0.2% (95%-CI: 0.1–0.3) for at least one of the specific alarm symptoms that led to a GP contact, respectively (Table 3).

**Table 3:** Positive predictive values and positive likelihood ratios for upper GI cancers divided by specific and non-specific upper GI cancer alarm symptoms experienced in the general population

Cases of upper GI cancers, numbers (N, %), positive predictive values, PPV (%) with 95%-CI and positive likelihood ratios (LR+)		Total
At least one alarm symptom	N (%)	12 (66.7%)
	PPV	0.1
	95% CI	(0.0;0.1)
	LR+	1.4
	95% CI(LR+)	(1.0;1.9)
At least one specific alarm symptom	N (%)	6 (33.3%)
	PPV	0.2
	95% CI	(0.1;0.5)
	LR+	4.4
	95% CI(LR+)	(0.7;1.7)
At least one non-specific alarm symptom	N (%)	9 (50.0%)
	PPV	0.1
	95% CI	(0.0;0.1)
	LR+	1.1
	95% CI(LR+)	(2.3;8.4)
Symptom experience and GP contact with at least one alarm symptom	N (%)	7 (38.9%)
	PPV	0.2
	95% CI	(0.1;0.3
	LR+	2.8
	95% CI(LR)	(1.6;5.0)

The LR+ for the association between symptoms and upper GI cancer are given in Table 3, Individuals experiencing a specific alarm symptom had a LR+ of 4.4 for being diagnosed with upper GI cancer, whereas individuals experiencing a non-specific symptom had a LR+ of 1.1.

Of the 18 individuals diagnosed with upper GI cancer, 66.7% had experienced at least one of the nine alarm symptoms, while 33.3% had experienced at least one of the four specific alarm symptoms.

In total, 38.9% of the individuals diagnosed with upper GI cancer reported that they had contacted the GP with at least one of the nine symptoms.

## Discussion

### Main findings

In this study we investigated the PPVs of specific and non-specific alarm symptoms of upper GI cancer in the general population. The PPVs were generally very low; the highest PPV was found among individuals who experienced at least one specific alarm symptom (0.2%) and among individuals who contacted the GP with at least one alarm symptom (0.2%). In total, 66.7% of the individuals diagnosed with upper GI cancer had reported experiencing at least one alarm symptom in the 12-month period prior to diagnoses. The LR+ was substantially higher for individuals experiencing a specific alarm symptom (LR+ 4.4), whereas experiencing a non-specific alarm symptom was not associated with upper GI cancer (LR+ 1.1).

### Strengths and limitations

The major strength of this study is the prospective cohort design, which gives the opportunity to retrieve information about symptom experiences prior to diagnosis. Using this study design we minimized the risk of recall bias that is often a challenge in studies regarding pre-diagnostic symptoms among cancer patients. Using register-based diagnoses rather than asking the respondents further reduces the risk of recall bias. The DCR was used to identify cases of cancer. This registry is based on mandatory data from several sources and is considered to be quite accurate [11].

In order to evaluate the relationship between symptoms and subsequent cancer diagnosis, we chose to include all upper GI cancers diagnosed in a time period of 12 months after reporting one or more of the alarm symptoms. We chose this time limit in order to enhance the likelihood of the symptom being linked to an underlying cancer. However, longer follow-up period might have contributed to a larger number of incident cancers, but we believe that increasing the time limit would weaken the linkage between symptom experience and subsequent diagnosis.

A general weakness of questionnaire-based studies is that respondents may not interpret the questions and categories of answers as intended. Prior to the survey, we conducted several rounds of pilot testing and field testing to reduce this possibility [9]. Based on the results of the pilot testing, it seemed reasonable to assume that the respondents understood the questions as intended.

This study reflects self-reported experience of symptoms and subsequent contacts with a GP. We asked whether the symptom was experienced within the preceding four weeks. Although we asked for symptom experiences and GP contacts within a short time period, some memory decay cannot be ruled out. The invitational letter for the questionnaire stated that this was a survey regarding symptoms, signs and healthcare seeking. A certain amount of social desirability bias cannot be ruled out, given that respondents may have felt that they should report a GP contact about an alarm symptom.

Another limitation is that we do not know if the symptom was a one-time experience that quickly resolved, or an ongoing/persistent symptom. The likelihood of the alarm symptom being caused by malignancy is likely to be very small if the symptom resolves spontaneously.

Another limitation is that more respondents were females and had a higher socioeconomic status compared to non-responders. It is well-known that persons with a lower risk of e.g. cancer are more likely to participate in surveys and even in cancer screening programs [13]. In our study it could have biased the results if the incidence of upper GI cancers had differed between responders and non-response. However, the incidence was similar in the two groups.

Since the incidence of upper GI cancer is relatively low in Denmark with only 18 cases of upper GI cancer in the 12-month follow up, it would have been preferable to estimate PPVs for each of the symptoms separately. However, this was not possible due to the size of the dataset according to Danish regulation. Using the absolute measure of PPV we cannot rule out that some associations were overlooked due to lack of power. Therefore, we chose to include the relative measure LR+ as well to enlighten the strength of association between the symptoms and cancer.

### Comparison with existing literature

The sensitivity and specificity of upper GI cancer alarm symptoms as well as the LR+ of the alarm symptoms have previously been demonstrated to be low among primary care patients [7] as well as in mixed cohorts from primary and secondary health care [14, 15]. In this study we confirmed that the predictive values of alarm symptoms of upper GI cancer in the general population are low. Based on existing knowledge about the diagnostic performance of symptoms indicative of upper GI cancer and the knowledge about how common GI symptoms are [16], we expected that the symptoms would not exhibit strong specificity despite being denoted cancer alarm symptoms.

An important aspect likely to influence the predictive value of a symptom is the severity of the symptom. Neither this study nor other studies included symptom severity [7], though it is an important factor in the clinical setting to triage of patients for referral.

## Conclusions

We found that the PPVs for alarm symptoms of upper GI cancer are quite low. Furthermore, we found that patients diagnosed with an upper GI malignancy had a higher likelihood of having experienced a specific alarm symptom of upper GI cancer 12 months prior to diagnosis, whereas there was no association between upper GI cancer and experience of non-specific alarm symptoms.

The responder analysis showed that more respondents were females, married/living together, had a high education and income level and were attached to the labour market.

Future studies focusing on the predictive values of different combinations of alarm symptoms could provide further insights. However, given the infrequent diagnosis of upper GI cancer this would require studying an ever larger population.

Despite the fact that symptoms are not as precise as a diagnostic tool such as e.g. laboratory data, because of their subjective and individual nature, quantifying the association between symptoms and upper GI cancer and comparing the relative diagnostic values is useful knowledge for the clinician.

Similarly, the results are helpful for health service planning. As the prognosis of upper GI cancer depends on stage at diagnosis, a logical instrument to improve survival could be to liberalise criteria for endoscopy. With more liberalised criteria for endoscopy some cancers could be detected earlier. However, given the low predictive value of specific and non-specific alarm symptoms it would result in considerable clinical and economic consequences and campaigns to increase awareness of symptoms of upper GI cancer have failed to demonstrate a significant impact on detection rate [17]. Furthermore, referring more patients for fast track investigations of symptoms that could be signs of cancer, but could just as well be due to other causes than cancer, could have harmful consequences [18, 19].

The findings of this study do not give rise to changing current guidance for referral for possible cancer. However, some symptoms such as nausea and abdominal pain are associated with low risk of upper GI cancer; hence these symptoms can be classified as “low risk but not no risk” symptoms [20].

In conclusion, new onset of alarm symptoms of upper GI cancers shows low predictive value for a diagnosis of upper GI cancer within the next year. Highest PPVs were found among individuals who reported experiencing specific alarm symptoms and among individuals who decided to consult their GP regarding alarm symptoms indicative of upper GI cancer. For the GP it is useful to know that despite experiencing alarm symptoms the risk of actually having upper GI cancer is low. This can among others be used in the communication with patients referred for fast track endoscopy. It is possible that being aware of the fact that alarm symptoms most often are not associated with upper GI cancer could reduce the anxiety known to be related to undergoing diagnostic evaluation for possible cancer [21].

## Abbreviations

CRS: Civil Registration System
DCR: Danish Cancer Registry
GI: Gastrointestinal
GP: General practitioner
LR+: Positive likelihood ratio
PPV: Positive predictive value
